# Adolescents' Use of Digital Technologies and Preferences for Mobile Health Coaching in Public Mental Health Settings

**DOI:** 10.3389/fpubh.2019.00178

**Published:** 2019-07-02

**Authors:** Kelly A. Aschbrenner, John A. Naslund, Elizabeth F. Tomlinson, Allison Kinney, Sarah I. Pratt, Mary F. Brunette

**Affiliations:** ^1^Department of Psychiatry, Geisel School of Medicine, Dartmouth College, Lebanon, NH, United States; ^2^Dartmouth-Hitchcock Clinic, Merrimack, NH, United States; ^3^Department of Global Health and Social Medicine, Harvard Medical School, Boston, MA, United States; ^4^Harvard Medical School, McLean Hospital, Belmont, MA, United States

**Keywords:** digital health interventions, adolescents, mental illness, health promotion, mobile health coaching, peer-to-peer support, social media

## Abstract

**Objective:** Youth with mental illnesses often engage in unhealthy behaviors associated with early mortality from physical diseases in adulthood, but interventions to support positive health behaviors are rarely offered as part of routine mental health care for this group. Digital health technology that is desirable, accessible, and affordable has the potential to address health behaviors in public mental health settings where many adolescents with severe mental health problems receive care. The aims of this study were to examine how adolescents receiving public mental health services use digital technology and social media and to explore their preferences using technology to support health and wellness.

**Methods:** Using a convergent parallel mixed methods design, we surveyed adolescents ages 13–18 from four community mental health centers in one state and conducted focus group interviews to explore their perspectives on using digital technology and social media to receive health coaching and connect with peers to support healthy behaviors. The survey and focus group data were merged to inform the future development of a digital health intervention for adolescents receiving public mental health services.

**Results:** Of 121 survey respondents (mean age 15.2, *SD* = 1.5), 92% had a cell phone, 79% had a smartphone, 90% used text messaging, and 98% used social media. Focus group interviews revealed that adolescents were interested in receiving strengths-based mobile health coaching, and they preferred structured online peer-to-peer interactions in which a professional moderator promotes positive connections and adherence to privacy guidelines.

**Conclusions:** Adolescents receiving public mental health services in this study had access to smartphones and were frequent social media users. These data suggest that digital health interventions to promote health and wellness among adolescents may be scalable in community mental health settings. Adolescent participants suggested that digital health interventions for this group should focus on strengths and online peer support for health promotion should include a professional moderator to foster and manage peer-to-peer interactions.

## Introduction

Improving the health and mental health of adolescents with serious psychiatric disorders, such as anxiety disorders, bipolar disorders, and psychotic disorders is a public health priority. Adults who have a serious mental illness (SMI) die 10 to 30 years earlier than the general population. These premature deaths are largely due to preventable and modifiable physical health conditions such as diabetes and heart disease. Approximately 50% of adults with SMI smoke (1), nearly two-thirds of individuals with schizophrenia are obese (2), and adults with SMI are significantly more likely to have low physical activity and cardiorespiratory fitness than people without mental illness regardless of socioeconomic status (3). Early intervention may be the key to preventing these dangerous health-risk behaviors before they cause long-term damage for individuals with mental illness. Although approximately 50% of all lifetime cases of mental illness begin to develop by the mid-teens and 75% by the mid-20s (4), few evidence-based strategies exist that specifically target health promotion in youth mental health service users, particularly among those who receive care in the public mental health sector that serve many poor and low income households.

National surveys show that digital technologies (e.g., use of the Internet, smartphones, mobile apps, and social media platforms) are integral to the lives of youth and young adults in the general population (5–7). The vast majority (88%) of US adolescents ages 13 to 17 have access to a personal computer or laptop at home, 95% of adolescents have access to a smartphone, 85% use social media, and 45% of teens describe their internet use as “near constant” (6). A large survey exploring how teens use digital technologies to access health information found that 84% of 1,156 teens reported getting health information online at least once in their lifetime (7). Among all teens, 42% had searched for fitness and exercise information online and 19% had searched for information on stress and anxiety. Digital technology has been studied as a way to deliver sexual health education (8), weight management (9–11), physical activity (12–14), and nutrition interventions (15, 16), and to enhance medication adherence (17) among adolescents in the general population. Several early studies have highlighted the promise of using technology to address adolescent mental health, including suicide prevention and outreach (18, 19) and treatment for depression and anxiety (20). However, to date there have been few targeted surveys of digital technology and social media use and preferences among the disadvantaged group of adolescents with mental illness who receive public mental health care to guide intervention development in this area.

Supported by digital technology, mobile health coaches could extend the reach of traditional public mental health services by delivering health promotion and prevention interventions for adolescents with mental illness. Health coaches embedded in routine mental health settings to deliver in-person lifestyle interventions for adults with SMI are effective at reducing cardiovascular risk in this population (21–23). Adapting evidence-based lifestyle intervention strategies and techniques to meet the unique needs of adolescents in public mental health settings by using digital technology, for example, smartphone-enabled health coaches, may expand the reach of these critical services particularly in under-resourced mental health care environments.

An important first step in assessing the potential to use digital technology to deliver health and wellness interventions to adolescents in the public mental health sector is to understand how this group is currently using technology and their experiences and preferences for using it. Recent studies have shown an association between social media use and mental health symptoms such as anxiety and depression in adolescents and young adults (24, 25). Other studies have found that social media use may not be a predictor of poor mental health in young people (26). Few, if any, studies have explored how adolescents receiving public mental health services use digital technology, including social media, and their preferences for using it to support health and wellness.

Experts agree that the effective design of digital health interventions requires a user-centered and iterative approach (27, 28), particularly for adolescents who are often early adopters of digital technology (11, 29). The aims of this study were to examine how adolescents receiving public mental health services use digital technology and social media and to explore their preferences for using technology to support health and wellness. We conducted a teen technology and social media use survey in public mental health settings and invited adolescents to participate in focus group interviews seeking input on intervention development.

## Materials and Methods

### Participants

Participants were adolescents ages 13 to 18 years old recruited from four community mental health centers (CMHCs) in one state in the Northeastern U.S. between March 2018 and January 2019. Adolescents were eligible to participate in the study if they were in the identified age range and received services at a participating mental health center. The Teen Technology and Social Media Use Survey was advertised on posters and brochures distributed by clinical staff at each CMHC. Prior to answering survey questions, the adolescent and a parent/legal guardian were prompted to read an information sheet (online or on paper) requesting permission from the parent/legal guardian and assent from the adolescent to complete the survey. Adolescents were asked to indicate their agreement to complete the survey with permission from a parent or legal guardian by checking a box online or on the paper form. Permission from a parent/legal guardian was not needed for 18-year old participants. Participants were compensated with a $10 gift card by mail for completing the survey. The survey did not collect any personally identifiable information. To collect payment, participants had to enter their name, mailing address, and contact information either online using the web-based survey platform or on a separate sheet of paper included with the paper survey.

Clinicians helped to recruit adolescents for the focus group interviews held at two of the four CMHCs by approaching adolescent clients and their parents/legal guardians with a study brochure and describing the purpose of the focus group. With parent/guardian approval, the names and phone numbers of interested adolescents and their parents/guardians were then sent to the study coordinator. The study coordinator called the parent/guardian to review and discuss the study information sheet, answer questions, and to obtain permission for their adolescent son or daughter to participate in the focus group if the youth was interested. The study coordinator then spoke to the adolescent on the phone to discuss and review the same information, answer questions and confirm their interest in participating in the focus group. Adolescents received a $20 gift card as compensation for their participation in the focus group interviews. Study procedures were approved by the Committees for the Protection of Human Subjects at Dartmouth College and the New Hampshire Department of Health & Human Services.

### Design

We used a parallel convergent mixed methods study design in which the quantitative survey data and qualitative focus group data were collected at the same time, analyzed separately, and then merged to gain a more complete picture of how digital technology and social media could be used to support health and wellness among adolescents receiving care at CMHCs. The data were integrated through a process of triangulation where we considered where findings from each method agreed (converged), offered complementary information (complementary), and appeared to contradict each other (discrepancy).

### Materials

The Teen Technology and Social Media Use Survey was adapted from our prior research exploring how young and middle-aged adults with SMI who receive services at CMHCs use technology and social media (30, 31). The Teen Technology and Social Media Use Survey consists of 21 open-ended and multiple-choice questions designed to explore access to and use of technology and social media, adolescents' reasons for using social media, privacy concerns and negative experiences interacting with others on social media, and preferences for connecting with peers from the mental health center on a social media platform.

Most survey questions were closed-ended, asking respondents to pick one response from a list of available options (for example, “Do you use the Internet?”) or multiple responses from a list of responses (for example, “Please check all of the social media sites that you use.”). Likert scale questions were also used in the survey (for example, “In thinking about your use of social media, how much do you agree or disagree with the following…”). Open-ended questions provided the opportunity to give other responses (for example, “Please tell us about any other ways you connect to the Internet.”). Participants were asked to provide their gender, age, and race.

The research team developed a focus group interview guide for the study that explored adolescents' experiences and preferences for using technology to support health and wellness. The interview guide covered the following categories: (1) experiences using social media and text messaging; (2) experiences with and preferences for being physically active; (3) experiences with and preferences for working with a coach; and (4) input on the development of a technology-based coaching and peer support intervention to support health and wellness.

### Procedures

To facilitate ease of participation among adolescent mental health service users, participants had the option of taking the anonymous survey online or on paper. To take the survey online, participants used the URL address listed on the survey brochure or scanned a barcode (also on the brochure) to open the survey on their smartphone or tablet. The web-based survey was created in Qualtrics, a HIPAA-compliant survey software licensed by Dartmouth College. Paper-based surveys included a stamped return envelope addressed to the research team.

The interviewer opened the focus group by explaining that the researchers planned to design a new program that uses technology to help teens who receive services at mental health centers connect with other teens to be more active, reduce stress, and look and feel better. The interviewer explained that the research team sought their thoughts and opinions about the type of program that might work best. The interviewer then presented the idea of using smartphones to receive mobile health coaching and a private social media group to connect with peers to support health promotion, and solicited participants' thoughts and opinions about this type of intervention. To clarify responses and explore answers in more depth during the focus group, the interviewer used follow-up questions and probes. The one-hour focus group interviews were audio-recorded and transcribed verbatim for data analysis.

### Data Analysis

#### Quantitative Data Analysis

Descriptive statistics were used to describe the age, gender, and race of the study sample, as well as their use of technology and social media. Frequencies and percentages were reported for categorical variables. Means and standard deviations (SDs) were used for continuous variables. SPSS version 25 was used to conduct quantitative data analysis.

#### Qualitative Data Analysis

We analyzed the qualitative data using thematic analysis, a method for identifying, analyzing, and reporting patterns within qualitative data (32). The qualitative data analyzed for the present study focused on adolescents' input on developing a technology-based coaching and peer support intervention to support health and wellness. The primary author (KA) developed a preliminary list of categories based on this broad topic. The second author (JN) coded the data using the preliminary code list as a guide, while editing and adding codes. The researchers selected representative quotes from the data for each category. Any disagreements were resolved through clarification and discussion. The final product was a list of major categories, subcategories, and representative quotes.

#### Data Integration

The data from the quantitative and qualitative analysis were triangulated to gain a more complete picture of adolescent preferences for online peer-to-peer support for health and wellness. The findings from each component of the study were laid out side by side. The team then identified where findings from the survey and focus group interviews agreed (converged), offered complementary information, and contradicted each other (discrepancy or dissonance).

## Results

During the 10-month survey period, 121 adolescents aged 13 to 18 responded to the survey at four participating CMHCs. The majority of respondents (85%) completed the survey online, either accessing the survey with the QR code or using the web link. Nineteen participants completed the survey on paper. The mean age of the respondents was 15.2 (*SD* = 1.5) and 59% were female. The majority of respondents identified as White (83%), followed by: Black (7%), American Indian (5%), and Asian (1%). Nine percent of respondents identified as Hispanic or Latino.

The respondents' digital technology and social media use survey data is summarized in Table 1. All 121 respondents used the Internet. Regarding digital technology use, 92% had a cell phone, 79% had a smartphone, 90% used text messaging, and 98% used social media. The most popular social media site used was YouTube. Seventy-two percent of respondents reported using social media several times per day or more. The top three reasons why respondents reported using social media was to keep in touch with family and friends (80%), to find information (63%), and to see what friends were doing (62%). Thirty two percent of respondents indicated they would be interested in connecting with other teenagers who go to the mental health center on a private social media group, while 33% were not interested, and 35% were unsure.

**Table 1:** Use of digital technology by adolescents receiving mental health services.

	***N***	**%**
Internet Use	121	100
**Top three ways connect to the Internet**		
Cell phone with internet access	111	92
Personal or family computer at home	95	79
Computer at school	79	65
**Technology ownership**		
Cell phone	112	93
Smartphone	96	79
A desktop or laptop computer	100	82
A tablet computer	51	42
Use cell phone to send and receive text messages	109	90
Use other device or program to send and receive text messages	71	59
Current social media use	119	98
**Top five media sites used (*****n=*** **119)**		
YouTube	107	90
Instagram	93	78
Snapchat	90	76
Facebook	80	67
Twitter	41	36
**Frequency of social media use (*****n=*** **119)**		
Never	2	2
A few times per year	0	0
Once or twice a month	3	2
At least once a week	7	6
Almost every day	22	18
Several times every day	87	72
**Frequency of social media interactions (e.g., posts, “likes,” or comments) (*****n=*** **119)**		
Never	11	9
A few times per year	7	6
Once or twice a month	12	10
At least once a week	28	23
Almost every day	32	26
Several times every day	31	26
**Time of day use social media the most (*****n=*** **119)**		
All the time	43	36
Morning	3	2
Afternoon	30	25
Evening/Night	28	24
After midnight	2	2
Don't know	15	13
**Number of friends/contacts on social media (*****n=*** **119)**		
Fewer than 50	28	23
51–100	14	12
101–200	7	6
More than 200	49	41
Don't know	23	19
**Top three reasons why use social media (*****n=*** **119)**		
To keep in touch with family and friends	95	80
To find information	75	63
To see what friends are doing	74	62
Use a fake social media account (e.g., Finsta)	30	25
**Interest in connecting with other teenagers who go to the mental health center on a private social media group**		
Yes	39	32
No	40	33
Don't know	42	35

### Qualitative Findings

Across two CMHCs, 15 adolescents (mean age = 15, *SD* = 1.0) participated in the focus group interviews. We identified categories reflecting participants' input on the development of a mobile health coaching and online peer support intervention to support health and wellness. Table 2 provides a summary of these major categories, codes, and representative quotes. Below, we describe each of the broad categories in greater detail.

1. **Strengths-based coaching**. Several participants mentioned that they would like help from a mobile health coach with goal setting and identifying aspirations, and that it was important to them that the coach focus on their strengths rather than their problems. Participants suggested that the mobile coach use challenges, rewards, and fun activities to generate interest in the intervention and motivate participants to reach their wellness goals.
2. **Peer and social connections**. Many participants wanted opportunities to meet new people, interact and socialize with others, and support one another's goals. Several participants wanted at least one in-person meeting with their mental health peers before joining them on a private social media group, and several participants expressed interest in access to an ongoing “optional” in-person meet-up throughout the intervention period.
3. **Staying safe online**. This was a prominent topic that emerged from the focus group discussions. Participants expressed concerns about privacy risks using social media to connect with peers at the mental health center, and a few were concerned about texting with a mobile health coach. Participants preferred that a coach or other professional moderate the peer-to-peer social media group to make sure the group was safe, and with whom they could share confidential information regarding any concerns about other participants. Several participants felt that it was important to meet the coach and other participants in person before interacting online.

**Table 2:** Thematic categories derived from adolescents' input on intervention development.

**Major categories**	**Codes for each category**	**Representative quotes from participants**
Strengths-based coaching	Help from mobile coach with identifying goals and aspirations Focus on success, not failures Incorporate challenges, rewards, and fun activities	*“I think it's [mobile health coaching] a cool idea, the concept…I like the idea of somebody who's not just there to help you get better, but to help you achieve things that you want to achieve and things that you enjoy.”* *“Exclusively positive challenges…I feel like not – not that like losing is bad, but like making it so if you don't reach your goal, you're not like, you're a failure.”* *“I think that would be a cool idea as long as it's not constantly talking about mental health. I want to have a good time, too… I just want connections with people, if you get what I mean.”* *“I guess a group challenge would be kind of cool. You kind of could see like maybe how much miles can you do in a week altogether.”*
Peer and social connections	Interact more with others Meet new people Social get-togethers Group support for meeting goals	*“I think it would be a good idea, because there is a lot of people that probably stay inside, and it will maybe encourage them to go outside and interact more.”* *“I think it would be cool to meet new people…I just like meeting new people. I have plenty of friends…but I wouldn't mind it.”* *“I feel like [in person peer group activities] should be required – like sometimes it should be required and sometimes it shouldn't. Sometimes if you're not achieving the goal the coach set, then you meet up together and wondering why you're not achieving it. And if you are [meeting goals], then it [meet ups] could be optional.”* *“If you're out [not achieving goals] you can still participate…just in a different way. That's not a negative or a positive, really. And I think that's kind of a happy medium to balance.”*
Staying safe online	Privacy concerns Professionally moderated social media group Meeting in person	*“That makes me feel anxious…Because social media can always be hacked, and that's just where I feel most anxious. I try to keep most of my conversations about what I really want to vent about with my friends, I don't do it over text. I wait until they come over…”* *“Personally, I wouldn't do this because I just wouldn't really feel comfortable talking to somebody online [referring to a mobile lifestyle coach], 'cause you never know. For me, it'd just be weird.”* *“Don't have anyone inappropriate [in the social media group], like people that do inappropriate stuff on social media, not have those people.”* *“So sometimes stuff flies around that shouldn't fly around, but if it's moderated then maybe [group members] would feel more comfortable, like talk about what they're doing. And it could help other people have ideas of what to do.”* *“If I met them [mobile health coach] maybe three or four times consecutive, then I probably would [use the intervention] because I'd get a feel for how they are, who they are. My counselor, I had to meet him numerous in order to even open up and talk.”* *“I thinking meeting in person [vs. entirely online social media group]…because there are things you can say in a group online, and then there's stuff you can say in person. It's like certain things you can explain better.”*

### Mixed Methods Findings

We merged results from the quantitative and qualitative data to generate insights into participants' interest and willingness to connect with other teenagers who receive services at the mental health center on a private social media group. Survey results indicated participants were almost evenly divided into three groups reflecting their interest in connecting with peers from the mental health center on a private social media group: 32% of respondents said yes, 33% were not interested, and 35% indicated they were unsure. The qualitative data indicated that although most participants saw potential value in connecting with peers online to support health and wellness, many participants had privacy concerns about using social media in this way and would want support, oversight, and reassurance from a professional to ensure safety in an online peer group. Several participants recommended there be an opportunity to meet the mobile health coach and peers in person before interacting with them on a private social media group. These qualitative data provide a more in-depth understanding of the survey results, in particular the reasons why over one-third of participants may have been unsure about connecting with peers online.

## Discussion

The present study contributes to the emerging literature on designing technologies for youth mental health by examining how adolescent mental health service users use digital technologies (e.g., smartphones and social media) and by exploring their preferences regarding how technology and social media could be used to promote health and wellness. The unique contribution of this study to the existing literature is the focus on adolescents who receive public mental health services where the majority of poor and low-income families turn for mental health care. To date, this vulnerable group of adolescents has been largely overlooked in the research literature, despite the potential benefits of using accessible and affordable technologies to promote health in this group. We found that 92% of adolescents had a cell phone, 79% of respondents had a smartphone, 90% used text messaging, and 98% used social media. This is consistent with technology use found in national surveys of the general adolescent population (5, 6), and is an important finding because it supports the potential generalizability and scalability of technology-based interventions for adolescent mental health service users in the public mental health sector. With respect to preferences for using digital technology to promote health and wellness, focus group interviews revealed that adolescents were interested in receiving support from a mobile health coach who focused on their strengths, and they preferred a professionally moderated social media intervention with opportunities for structured online peer-to-peer interactions in which the moderator promotes positive connections and adherence to privacy guidelines.

The top three reasons why adolescents in our study reported that they used social media were: (1) to keep in touch with family members and friends; (2) to find information; and (3) to see what friends are doing. The benefits to adolescents of using online technologies, including social media, reported in the literature include increased self-esteem, perceived social support, increased social capital, safe identity experimentation and increased opportunity for self-disclosure (33). Psychiatric symptoms and behavioral problems associated with emotional disturbance in adolescence can make in-person social interactions challenging and forming and maintaining friendships difficult (34, 35). The asynchronous communication afforded by social media may offer novel opportunities for adolescents who experience disabling mental health symptoms to increase their confidence interacting and connecting with others and improve communication skills in the online context of their everyday life. For example, adolescents who struggle with social anxiety may experience less anxiety when interacting with peers online vs. offline, with online interactions allowing them time to practice social skills in what feels like a more controlled environment.

When presented with the idea of a mobile health coach to promote health and wellness, participants in this study indicated they would like help with goal setting and identifying aspirations, and that it was important to them that the coach focus on their strengths rather than their weaknesses. This perspective is consistent with prior research showing young adults' preferences for recovery-oriented mental health care that prioritizes autonomy, empowerment, and respect for the individual (36). Digital technology can be a powerful tool for delivering strengths-based coaching for health promotion and prevention to individuals with mental illness by enabling coaches to provide frequent, personalized messages that support and encourage progress toward health and wellness goals (37–39). Our team is currently evaluating a strengths-based mobile health technology and peer support intervention for reducing cardiometabolic risk among young adults ages 18 to 35 living with SMI and who are overweight and/or obese (31). There is significant untapped potential to leverage mobile health coaching as a strategy to prevent cardiometabolic risk factors such as obesity and smoking and subsequent poor health outcomes that are highly prevalent among adults with SMI. Technology-based interventions tailored for adolescents with mental health problems could target mechanisms, such as exercise, known to reduce cardiometabolic risk and improve mental health outcomes. In addition, evidence-based illness management and recovery programs designed to help adults with SMI manage their illness and achieve personal recovery goals could be adapted for adolescents with mental illness and delivered using technology as a prevention strategy for this high-risk group.

Mobile health coaching strategies will likely need to be tailored to meet the unique needs and preferences of a younger generation of mental health services users. For example, prior research on the use of text messaging to engage adolescents in health promotion has identified adolescents' preferences for text message content that is informative (e.g., relevant and new information), simple (e.g., use of small words and phrases) with a conversational tone, and sociable (e.g., non-stigmatizing and easily able to be shared with friends) (40, 41). Future research could explore in more depth, with feedback from adolescents receiving mental health services, how best to tailor digital health interventions that were designed for adults, including text messaging and social media content.

Participants in this study were highly active on social media; 91% used social media at least once per day and 40% reported more than 200 connections on social media sites. However, when asked in the survey if they were interested in connecting with other teenagers who receive treatment at the mental health center on a private social media group, 32% indicated yes, 33% were not interested, and 35% indicated they were unsure. Online peer-to-peer support has the potential to reduce stigma, promote social connectedness and ultimately, improve the well-being of people with mental illness (42). However, during qualitative interviews many participants in our study indicated they were concerned about the potential risks of joining peers from the mental health center on a social media group. Literature reviews suggest that cyberbullying may be more common among adolescents struggling with mental health problems (43–45). To prevent negative experiences with peers online, participants in this study recommended that the mobile health coach moderate the peer social media group and be a person to whom they could report confidential information regarding any concerns that arise about other participants. As social media is increasingly being used as a promising strategy for delivering health-related interventions to adolescents (15, 46–49), careful thought will need to be given to the role of a professional moderator who could foster prosocial peer interactions and prevent and address privacy concerns that may discourage adolescents from taking advantage of health promoting social media interventions.

### Study Limitations

Several limitations warrant consideration. First, we recognize that our findings should be interpreted cautiously given the small sample size. It is possible that the adolescents who were the most disinterested in using technology in this way did not participate in the focus group interviews and thus, their perspectives were not reflected in the study findings. Second, we recruited adolescents receiving treatment at CMHCs to participate in the survey and focus group interviews but we did not collect data on their psychiatric diagnoses. It is possible that adolescents with more severe psychiatric disorders were not able to complete the survey and engage in the focus group interviews. However, we conducted this study at safety net CMHCs that typically serve the most vulnerable adolescents with mental illness, thereby offering unique findings on an at-risk service user group. Using an online survey, we were able to survey 121 adolescents receiving care in under resourced public mental health centers with minimal burden on clinical staff and administrators. This low-cost online survey was administered in real world mental health clinics and thus demonstrated the feasibility of using online surveys to collect data from hard-to-reach populations including adolescents with mental illness. Digital technologies can be applied to assess and monitor psychiatric disorders (50), monitor alcohol and drug use (51) among adult populations. This study did not explore the participants' views on these specific applications of digital technology. Finally, there was limited racial and ethnic diversity in this sample. However, national reports on adolescent technology and social media use that have included diverse samples show results that are similar to ours (6).

## Conclusions

Our findings show that many adolescents receiving public mental health services in CMHCs have access to smartphones and are frequent social media users. This is an important finding because it highlights the promise of using digital health interventions in under resourced health care settings to intervene early to promote health and wellness among adolescents experiencing mental illness. Based on recommendations from adolescent participants in this study, digital technology interventions that use mobile health coaches to promote health and wellness should focus on strengths and online peer interactions should be moderated to foster and manage peer-to-peer interactions.

## Data Availability

The datasets generated for this study are available on request to the corresponding author.

## Ethics Statement

This study was carried out in accordance with the recommendations of the Committees for the Protection of Human Subjects at Dartmouth College (CPHS #30817) and the New Hampshire Department of Health & Human Services (CPHS# 258) and approved by these committees. All subjects gave informed consent (adults) and assent (adolescents) in accordance with the Declaration of Helsinki.

## Author Contributions

KA, JN, and ET conceived, designed, and wrote the protocol for this study. AK was responsible for coordinating the study and the acquisition of the data. MB and SP reviewed the first draft of the manuscript and contributed to the sections of the manuscript. All authors provided critical review of the manuscript and approved the final version.

### Conflict of Interest Statement

The authors declare that the research was conducted in the absence of any commercial or financial relationships that could be construed as a potential conflict of interest.

## Abbreviations

SMI: serious mental illness
CMHCs: community mental health centers.
